# Latent class distributional regression for the estimation of non-linear reference limits from contaminated data sources

**DOI:** 10.1186/s12859-020-03853-3

**Published:** 2020-11-13

**Authors:** Tobias Hepp, Jakob Zierk, Manfred Rauh, Markus Metzler, Andreas Mayr

**Affiliations:** 1 Institut für Medizininformatik, Biometrie und Epidemiologie, Friedrich-Alexander-Universität Erlangen-Nürnberg, Waldstraße 6, 91054 Erlangen, Germany; 2 Kinder- und Jugendklinik, Universitätsklinikum Erlangen, Loschgestraße 15, 91054 Erlangen, Germany; 3 Institut für Medizinische Biometrie, Informatik und Epidemiologie, Universitätsklinikum Bonn, Venusberg-Campus 1, 53127 Bonn, Germany

**Keywords:** Latent class regression, Finite mixture models, Distributional regression, Reference limits

## Abstract

**Background:**

Medical decision making based on quantitative test results depends on reliable reference intervals, which represent the range of physiological test results in a healthy population. Current methods for the estimation of reference limits focus either on modelling the age-dependent dynamics of different analytes directly in a prospective setting or the extraction of independent distributions from contaminated data sources, e.g. data with latent heterogeneity due to unlabeled pathologic cases. In this article, we propose a new method to estimate indirect reference limits with non-linear dependencies on covariates from contaminated datasets by combining the framework of mixture models and distributional regression.

**Results:**

Simulation results based on mixtures of Gaussian and gamma distributions suggest accurate approximation of the true quantiles that improves with increasing sample size and decreasing overlap between the mixture components. Due to the high flexibility of the framework, initialization of the algorithm requires careful considerations regarding appropriate starting weights. Estimated quantiles from the extracted distribution of healthy hemoglobin concentration in boys and girls provide clinically useful pediatric reference limits similar to solutions obtained using different approaches which require more samples and are computationally more expensive.

**Conclusions:**

Latent class distributional regression models represent the first method to estimate indirect non-linear reference limits from a single model fit, but the general scope of applications can be extended to other scenarios with latent heterogeneity.

## Background

Reference intervals play an important role in clinical practice in deciding whether a particular test result measured in a patient should be considered physiological or pathologic. As a consequence, proper determination of the reference limits, i.e. the bounds that define these intervals, has been extensively discussed in the recent decades, leading to the proposition of several guidelines [1, 2]. Although prospective approaches using only samples from healthy individuals from the reference population are often considered the gold standard for reference interval determination [3], they require comprehensive and careful definition of the inclusion criteria and recruitment of an appropriate reference population. Together with the recommendations that each laboratory should establish its own reference intervals due to potential transferability problems and conduct periodical reviews of the resulting estimates, this gold standard is an enormous and unmet challenge for most laboratories [4].

This task becomes even more demanding considering that many analytes vary greatly with respect to different covariates of the patient [5]. In practice, this problem is often solved by splitting the population into subgroups to determine separate intervals. While this approach seems reasonable for categorical features such as sex, the decision on how to define the cutpoints to differentiate between multiple age groups is substantially more challenging. Discretization inevitably leads to discontinuities in the reference limits and thus again to increasing uncertainties near these cutpoints. Early work by Virtanen et al. [6] already proposes regression based estimation to address this issue, but the applied models are rather limited and not sufficient to describe the full conditional distribution of most analytes.

Given that age-dependent variations are particularly pronounced during childhood, however, it can be difficult to reach sample sizes big enough to establish reliable intervals over the whole age range, as the recruitment of children for medical studies is subject to strict regulations [7]. Alternative solutions therefore rely on the retrospective use of existing data from laboratory databases to estimate “indirect” reference intervals [4]. However, these databases are contaminated in the sense that it is rarely possible to reconstruct the analyte-specific health status for most entries, as the sampled individuals have not undergone a screening process comparable to the strict inclusion criteria mentioned above, but laboratory testing was performed as part of a diagnostic workup. As a consequence, the retrospective estimation of reference intervals requires additional precautions in order to avoid corruption by pathological samples. While several adequate methods based on statistical procedures have been developed to decompose the unlabeled empirical distribution, they mainly focus on estimating reference intervals independently from covariates.

In this article, we suggest a new approach to derive reference limits from clinical laboratory databases using mixtures of Gaussian location and scale models via the framework of generalized additive models for location, scale and shape (GAMLSS) [8]. The general framework of GAMLSS and other variants of distributional regression have a long history in the construction of reference growth charts for children [9–11] and are particularly recommended by the WHO for this task [12]. However, these classical approaches rely on data from reference populations and hence are not directly suitable for our data situation with latent classes (for a Markov-switching GAMLSS approach for latent *states* see [13]).

By incorporating mixtures of GAMLSS to simultaneously model the non-linear dependence structure of both mean and variance parameter for the unknown healthy and non-healthy components, our new approach allows for a very flexible solution to estimate the underlying distributions of reference limits from heterogeneous but unlabeled data sources. In contrast to recent contributions addressing these issues by splitting the data into multiple overlapping windows and subsequently interpolating the individually calculated reference limits [14, 15], our solution provides an integrated approach that only requires a single fit to the data. As a consequence, latent class distributional regression represents the first approach capable of simultaneously accounting for both non-linear dependencies of covariates with respect to multiple distribution parameters and unlabeled data sources in this setting. We demonstrate our approach by estimating continuous hemoglobin reference intervals for boys and girls in a heavily contaminated real-world dataset from a tertiary care center.

## Results

### Simulation study

To investigate whether or not the suggested latent class distributional regression models are able to approximate the true underlying non-linear components of an unlabeled dataset, we examined the performance of our approach using an adaptive simulation scenario described in this section. With the regressor variables $\varvec{x}$ standard uniformly distributed, all responses were sampled from a Gaussian mixture with two components as follows:

$$\begin{aligned} \varvec{y}\sim \alpha _1{\mathcal {N}}\left( \boldsymbol{\eta }_{1\mu }, \boldsymbol{\eta }_{1\sigma }^2\right) +\alpha _2{\mathcal {N}} \left( \boldsymbol{\eta }_{2\mu },\boldsymbol{\eta }_{2\sigma }^{2}\right) \end{aligned}$$

The first component in each scenario is considered to be the distribution of main interest, i.e. the distribution of what will later be attributed to the “healthy” part of the sample, with

$$\begin{aligned} \boldsymbol{\eta }_{1\mu } = \varvec{x}+10\sin \left( \frac{\left( \varvec{x} -0.5\right) \sqrt{12}\pi }{2}\right) \end{aligned}$$

and

$$\begin{aligned} \boldsymbol{\eta }_{1\sigma } = \exp \left( 8+5\varvec{x}\right) . \end{aligned}$$

In order to be able to evaluate the performance of the algorithm under varying conditions, an adjustable specification was considered for the second component to account for different degrees of overlap and the resulting distinctiveness between the true distributions. This was achieved by using an additional spacing variable *c* for the mean formula:

$$\begin{aligned} \boldsymbol{\eta }_{2\mu } = c+c\varvec{x}+10\sin \left( \frac{\left( \varvec{x}-0.5\right) \sqrt{12}\pi }{2}\right) \end{aligned}$$

With

$$\begin{aligned} \boldsymbol{\eta }_{2\sigma } = \exp \left( 11+9\varvec{x}\right) , \end{aligned}$$

the variance of the second component is not affected by *c* and hence the same for all simulation settings. Consequently, the smaller *c* the larger the overlap, with expected values identical but $\eta _{1\sigma }^{(i)} < \eta _{2\sigma }^{(i)}$ for all *i* if *c* = 0. In order to further diversify the simulation setup, we additionally considered different mixture weights $\boldsymbol{\alpha }$ to reflect different amounts of contamination in the data, with $\alpha _1$ always denoting a more dominant “healthy” component.

**Fig. 1:**
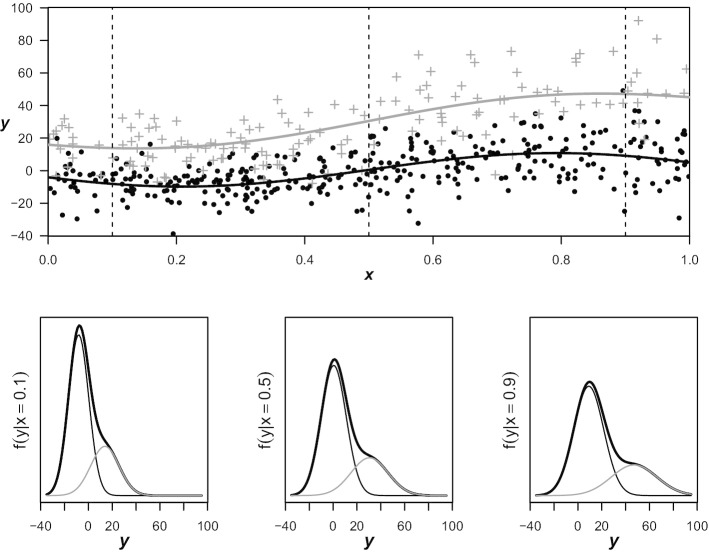
Simulation example. Exemplary data with *n* = 500 cases, gap parameter *c* = 20 and $\boldsymbol{\alpha }=(0.7,0.3)$. Figures in the bottom row show the true mixture and component densities for $x\in (0.1,0.5,0.9)$

Figure 1 shows an exemplary simulated data set, where it can be seen that the locations of the two distributions slowly diverge and their variances increase for larger values of $\varvec{x}$. Revisiting the details of the true components given above, it is clear that the exponent of both variance terms is actually even linearly increasing with respect to $\varvec{x}$. However, we decided to use non-linear prediction functions for all four distribution parameters, standard deviations included. First, because we felt that this form of linearity might not only be difficult to detect but would also be rarely considered appropriate in most practical applications, and second, because it allowed us to simultaneously investigate how the algorithm deals with over-specification of the model. Parameter estimation regarding the different components of the mixture distribution was achieved using the maximum likelihood approach implemented in the $\texttt {gamlss}$-package for $\texttt {R}$ [16, 17] wrapped inside an EM-algorithm as described in Algorithm 1 with the convergence threshold $\epsilon$ set to 0.001. Both non-linear terms for each component were estimated using cubic B-splines with three degrees of freedom. A reproducible capsule of the $\texttt {R}$-code used for data generation and analysis is publicly available on Code Ocean [18].

While many applications of mixture models are initialized using e.g. random assignment of observations, doing so resulted in many unreasonable solutions, especially for higher values of *c*. This was a result of the algorithm quickly running into local maxima due to its high flexibility, especially with respect to the variance components. Therefore, we addressed this issue by providing more informative initial weights based on the cumulative distribution function derived from a somewhat ‘naive’ application of a Gaussian GAMLSS that ignores the fact that the data is sampled from a mixture.

Overall, our results are based on 24,000 independently generated datasets, i.e. 1000 repetitions for each unique combination of $c\in (5,10,15,20)$, $\alpha _1\in (0.6,0.7,0.8)$ and $n\in (500,1000)$, specifically. In 4.43% of all runs the algorithm encountered problems before reaching the targeted convergence threshold of $\epsilon =0.001$. About 43% of these issues occurred when *c* was set to 5, which is not that surprising considering the substantial overlap between both components in these rather extreme scenarios. Interestingly, a feasible solution in most of these cases was to rerun the algorithm with randomly assigned component memberships as starting weights, as this resulted in one component to quickly take over and explain large parts of the data, while the second addressed the most extreme outliers. Although the affected runs did not necessarily result in obviously wrong estimations of the desired quantiles when using the naive model as initialization, we decided to omit them from the aggregated results to base the comparisons on identical conditions with respect to the initialization strategy. For a more detailed breakdown, see Table 3 in the “Appendix”.

Along with the results of the latent class distributional regression model (LCDR), we additionally report the performance of two standard GAMLSS fits. The first is the “naive” fit to the full data set used for the initialization of the LCDR, which ignores the true nature of the data and should therefore perform rather poorly. The second is a GAMLSS fit only to those observations in each simulated dataset that are in fact sampled from the “healthy” first component. This model can be considered as the gold standard that could have been achieved with a prospective study design. Main objective of all three models is the estimation of ${Q}_{1,0.95}(\varvec{x})$, i.e. the 95%-quantile of the first component.

Tables 1 and 2 show the aggregated results of the three models, i.e. latent class distributional regression (LCDR), “gold standard” (GOLD) and “naive” fit (NAIVE), for each scenario with *n* = 500 and *n* = 1000, respectively. With the predictor variable $\varvec{x}$ standard uniformly distributed, we calculate the integrated error (IE) $\int _0^1 \hat{Q}_{1,0.95}(x)-{Q}_{1,0.95}(x)\,dx$ as a measure of bias together with the integrated squared error (ISE) $\int _0^1 (\hat{Q}_{1,0.95}(x)-{Q}_{1,0.95}(x))^2\,dx$ as a measure of general deviation from the true quantile for each run.

**Table 1:** Simulation results for *n* = 500

$\alpha _1$	*c*	LCDR		GOLD		NAIVE	
*Integrated error*							
0.6	20	− 0.185	(2.837)	− 0.160	(0.942)	26.60	(1.223)
	15	− 0.318	(3.365)	− 0.172	(0.936)	19.33	(1.160)
	10	− 0.924	(3.753)	− 0.169	(0.945)	12.81	(1.087)
	5	− 1.997	(3.897)	− 0.179	(0.936)	7.410	(0.980)
0.7	20	− 0.496	(2.528)	− 0.110	(0.863)	21.86	(1.225)
	15	− 0.711	(2.885)	− 0.106	(0.859)	15.66	(1.146)
	10	− 1.596	(3.297)	− 0.090	(0.858)	10.19	(1.058)
	5	− 2.895	(3.810)	− 0.103	(0.858)	5.760	(0.958)
0.8	20	− 0.563	(2.168)	− 0.082	(0.821)	16.18	(1.205)
	15	− 0.991	(2.459)	− 0.089	(0.824)	11.40	(1.129)
	10	− 2.112	(2.943)	− 0.064	(0.825)	7.243	(1.030)
	5	− 3.599	(3.645)	− 0.062	(0.825)	3.967	(0.921)
*Integrated squared error*							
0.6	20	17.05	(17.81)	3.792	(2.770)	747.43	(74.07)
	15	22.15	(26.54)	3.804	(2.788)	397.42	(50.93)
	10	28.59	(39.72)	3.843	(2.799)	177.19	(31.74)
	5	42.32	(57.01)	3.788	(2.759)	61.99	(16.77)
0.7	20	13.06	(11.57)	3.253	(2.325)	509.10	(61.96)
	15	16.31	(17.00)	3.250	(2.330)	264.13	(41.33)
	10	23.82	(29.69)	3.258	(2.352)	114.47	(24.89)
	5	41.57	(56.00)	3.238	(2.310)	39.07	(13.05)
0.8	20	9.919	(8.52)	2.869	(2.154)	284.09	(45.34)
	15	13.16	(12.49)	2.883	(2.183)	143.68	(29.81)
	10	21.86	(26.45)	2.883	(2.168)	60.38	(17.29)
	5	45.80	(58.75)	2.880	(2.176)	20.39	(8.814)

Reported are the means and standard deviations of the integrated error and the integrated squared error over all simulation runs for latent class distributional regression (LCDR), “gold standard” (GOLD) and “naive” fit (NAIVE)

**Table 2:** Simulation results for *n* = 1000

$\alpha _1$	*c*	LCDR		GOLD		NAIVE	
*Integrated error*							
0.6	20	− 0.200	(2.110)	− 0.059	(0.652)	26.66	(0.872)
	15	− 0.218	(2.601)	− 0.060	(0.651)	19.38	(0.828)
	10	− 0.309	(2.755)	− 0.056	(0.653)	12.87	(0.773)
	5	− 0.987	(3.057)	− 0.057	(0.656)	7.501	(0.706)
0.7	20	− 0.436	(1.876)	− 0.056	(0.594)	21.92	(0.871)
	15	− 0.482	(2.183)	− 0.057	(0.594)	15.72	(0.825)
	10	− 0.680	(2.314)	− 0.061	(0.594)	10.23	(0.764)
	5	− 1.621	(2.53)	− 0.063	(0.594)	5.808	(0.689)
0.8	20	− 0.394	(1.637)	− 0.061	(0.565)	16.17	(0.845)
	15	− 0.540	(1.908)	− 0.054	(0.568)	11.42	(0.793)
	10	− 1.010	(2.022)	− 0.057	(0.570)	7.263	(0.733)
	5	− 2.199	(2.560)	− 0.050	(0.570)	4.009	(0.660)
*Integrated squared error*							
0.6	20	8.326	(8.142)	1.910	(1.378)	746.50	(52.97)
	15	11.69	(14.89)	1.911	(1.380)	396.38	(36.42)
	10	13.47	(17.26)	1.913	(1.383)	176.55	(22.58)
	5	19.92	(28.98)	1.914	(1.384)	61.46	(12.09)
0.7	20	6.764	(5.920)	1.692	(1.185)	506.36	(43.79)
	15	8.532	(7.778)	1.694	(1.187)	262.09	(29.60)
	10	10.02	(10.58)	1.692	(1.186)	112.57	(17.90)
	5	17.01	(25.73)	1.685	(1.181)	37.64	(9.275)
0.8	20	5.150	(4.574)	1.495	(1.007)	279.49	(32.28)
	15	6.634	(5.876)	1.494	(1.009)	140.29	(21.19)
	10	8.354	(9.089)	1.494	(1.003)	58.09	(12.47)
	5	19.34	(27.67)	1.500	(1.010)	18.94	(6.266)

Reported are the means and standard deviations of the integrated error and integrated squared error over all simulation runs for latent class distributional regression (LCDR), “gold standard” (GOLD) and “naive” fit (NAIVE)

As should be expected, the LCDR is mostly situated between the two GAMLSS fits. With increasing values of *c*, the underlying components are easier to differentiate and LCDR performance generally increases, while the naive model deteriorates. Note that the results for the gold standard models only vary with respect to different *c* because of the runs removed due to non-convergence of the LCDR-algorithm on the same dataset.

While outperforming the naive approach in every setup, the LCDR shows a tendency to underestimate the true 95%-quantiles for very low *c*. This can be traced back to initializing the models in a way that they start far away from each other. If the overlap of the mixture components is very large, it is harder for the algorithm to bring the components close enough together while simultaneously trying to estimate their shape, whereas smaller overlap means more favorable and convenient starting positions.

**Fig. 2:**
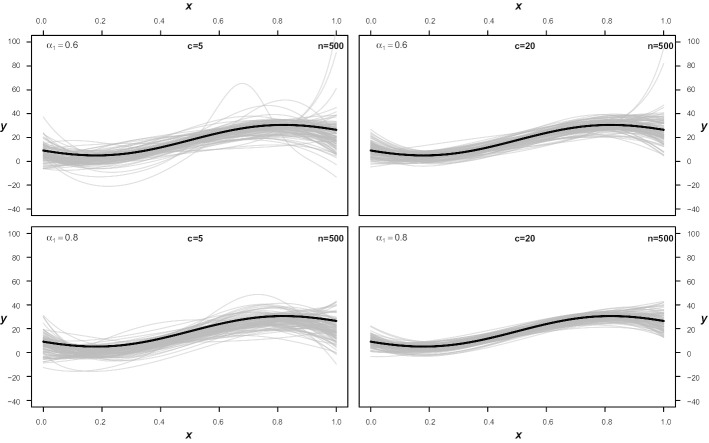
Estimated 95%-quantiles with *n* = 500. Shown are the first 100 estimated quantiles from four different settings, where $\alpha$ is the proportion of observations sampled from the corresponding component and *c* is the amount of overlap as described in the simulation section

**Fig. 3:**
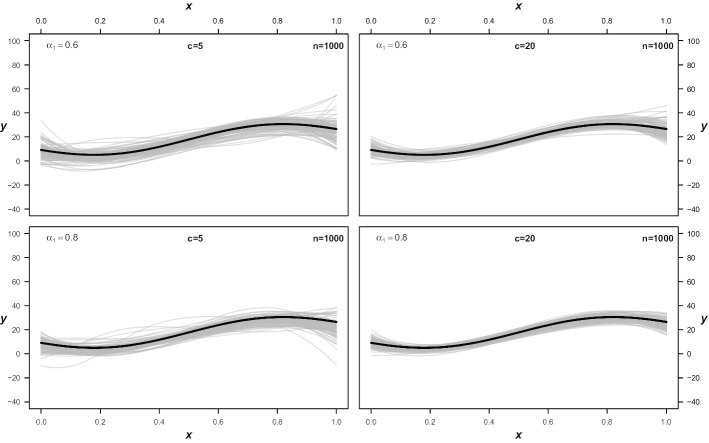
Estimated 95%-quantiles with *n* = 1000. Shown are the first 100 estimated quantiles from four different settings, where $\alpha$ is the proportion of observations sampled from the corresponding component and *c* is the amount of overlap as described in the simulation section

The integrated squared error further affirms this issue. With increasing sample size, however, the average ISE of the LCDR improves considerably. Figures 2 and 3 show the quantiles estimated from the first 100 generated datasets for four corresponding scenarios. While a few estimated limits deviate heavily from the true quantile in the settings with *n* = 500 especially at the boundaries of $\varvec{x}$, such obvious errors are no longer present when the sample size is doubled and the true quantile is much better approximated. Of course, this could have been easily prevented by using linear predictors for the variance parameters of the components or rerunning the algorithm with a new set of initial weights in the most obvious cases. Therefore, sample size plays an important role to avoid running into local maxima due to overfitting random patterns in the data.

In an additional simulation study involving a mixture of gamma distributions, the algorithm performs similarly well as in the Gaussian settings. However, the importance of a sufficiently large sample is also evident in these scenarios. The corresponding results are available in full detail on Code Ocean [18].

### Age-dependent hemoglobin reference intervals

We apply our model to the estimation of pediatric hemoglobin reference intervals. All laboratory tests were performed in the context of patient care in the Department of Pediatrics and Adolescence at the University Hospital Erlangen, Germany. We used test results from all children, irrespective of health status or speciality unit, including intensive care and oncology units.

**Fig. 4:**
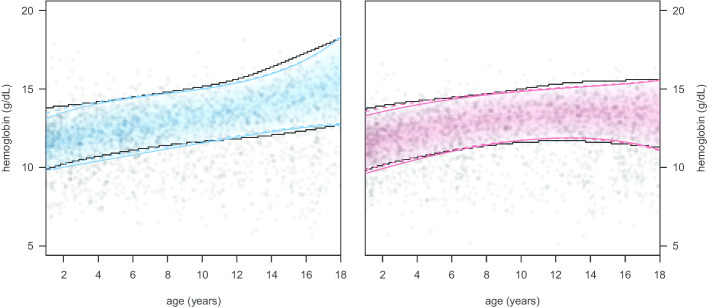
Reference intervals for hemoglobin concentration. Shaded areas represent the estimated distribution of healthy hemoglobin concentration enclosed by the 2.5% and 97.5% quantiles for boys (left) and girls (right) represented by colored solid lines, whereas the dashed lines result from fitting separate models for boys and girls. Black solid lines show solutions from an alternative approach estimated by splitting the population into multiple subgroups

After removing samples taken at subsequent visits of the same person, $n_f=60424$ and $n_m=75464$ observations of girls and boys aged between 1 and 18 years are available. The model is estimated as a mixture of two Gaussian distributions conditional on age and sex using

$$\begin{aligned} \boldsymbol{\eta }_{m\mu }&= \beta _{m0\mu } + \beta _{m1\mu }\cdot I_{female}(\mathbf{sex} )\\ &\quad +h_{m1\mu }\left( \mathbf{age} \cdot I_{female}(\mathbf{sex} )\right) \\ &\quad +h_{m2\mu }\left( \mathbf{age} \cdot I_{male}(\mathbf{sex} )\right) \\ \boldsymbol{\eta }_{m\sigma }&= \beta _{m0\sigma } + \beta _{m1\sigma } \cdot I_{female}(\mathbf{sex} ) \\ &\quad +h_{m1\sigma }\left( \mathbf{age} \cdot I_{female}(\mathbf{sex} )\right) \\ &\quad +h_{m2\sigma }\left( \mathbf{age} \cdot I_{male}(\mathbf{sex} )\right) \end{aligned}$$

for both $m \in (1,2)$ components, where $I(\cdot )$ is the indicator function. As in the simulations, we used B-splines with three degrees of freedom for all non-linear model terms. Figure 4 shows 5000 randomly selected data points from the full dataset. The shaded areas represent the estimated distribution for the healthy values of hemoglobin concentration enclosed by the 2.5% and 97.5% quantiles depicted by red or blue solid lines. Fitting the models separately to the data from boys and girls with age as single non-linear predictor led to almost identical quantiles depicted by dashed lines. The solid black lines show the corresponding quantiles based on estimations available from an alternative approach, that has been evaluated extensively but requires splitting of the dataset and subsequent interpolation of discrete reference intervals. Overall, both methods provide very similar estimates.

## Discussion

In this article, we propose an algorithm that generally extends the framework of latent class regression models to settings with distributional response variables. We use the resulting model to estimate age-specific hemoglobin reference limits derived from laboratory databases containing unlabeled samples from both healthy and children with a wide range of diseases. While the framework is by no means limited to these specific scenarios and can be used for a large variety of research questions associated with unobserved heterogeneity, the proposed approach clearly presents a valuable contribution to the field of reference interval estimation, where methods capable of simultaneously accounting for both non-linear dependencies of covariates with respect to multiple distribution parameters and unlabeled data sources are still lacking.

The algorithm performs very promising in our simulation study even in the most difficult scenarios with extreme overlap between the components. Our simulation study had been motivated by the actual application, however, further research is warranted to extend the approach to more complex scenarios that have not been considered at the moment: First, we restricted our simulations to scenarios with two components, one for the healthy and another one for the pathologic samples. This is perfectly fine if the majority of pathologic values are either higher or lower compared to those considered healthy, as is the case in our application. For other analytes, it may be necessary to specify an additional pathologic distribution to cover deviations from the healthy range in both directions. Moreover, our simulations so far only focused on mixtures of Gaussian and gamma distributions, which may not always represent the best choices in all situations. However, both aspects can generally be addressed by increasing the overall number of components *M* and using different probability distribution functions for those components. Although the simulation study addresses only scenarios with a single predictor variable, the very similar solutions of the joint and separate models for boys and girls regarding pediatric hemoglobin reference limits further offer a promising perspective for expandability towards scenarios with more than one covariate.

Currently, the algorithm uses unpenalized B-splines to estimate the non-linear model terms. Therefore, the degree of smoothness of the effects has to be specified in advance, which will not always be a straightforward decision in practice. A natural expansion of the proposed algorithm is hence the introduction of a penalty to reduce the risk of overfitting. However, the model is already very flexible in its current implementation, which makes the solutions strongly depend on the initial observation weights. As a consequence, the stability of the suggested solutions of the algorithm should be further evaluated in practice by using multiple sets of different starting weights or bootstrapped data sets. Since straying into local maxima is strongly determined by the extent to which the first iterations pick up misleading patterns, an important extension would hence be the search for possibilities to absorb these early missteps. Reducing the dependence on the initialization can then extend the applicability of the model to situations in which theoretically meaningful assumptions about the class memberships are not available in advance. In the context of indirect reference interval estimation, introducing model constraints that prevent crossing of the location parameters could further guide the algorithm to avoid theoretically unreasonable solutions, especially if the model involves separate components for low and high pathologic measurements.

Currently, another limitation are the mixture weights $\boldsymbol{\alpha }$, which are estimated constant with respect to the values of the predictor variable(s). It is actually not entirely unlikely, however, that the mixture ratio of the components depends on some external factor as well. Regarding the values of analytes sampled from children, for example, the proportion of healthy samples may eventually be decreasing with age, as routine examinations become less frequent and many of the samples obtained in everyday care are taken from children who probably visit the hospital for reasons that may also affect the analyte of interest. Therefore, approaches that are able to estimate dynamic mixture weights as well could be a valuable extension of the model framework. Further possible improvements of our approach involve the implementation of lower bounds for certain distribution parameters like the Gaussian variance parameter to avoid potential problems with respect to unbounded likelihoods.

## Conclusion

Latent class distributional regression models provide a both practical and theoretically sound framework suitable for, but not limited to, the estimation of reference limits from contaminated databases. Due to the possibility to allow for very flexible model components, the application requires careful considerations from the researcher with respect to meaningful initial weight vectors and degrees of freedom for the non-linear effects. Moreover, these efforts should also be supported by an appropriately large sample in relation to the desired degree of flexibility.

## Methods

### Finite mixture models

Applying a (parametric) statistical model to a dataset usually requires the assumption of a probability density function that describes the distribution of the variable of interest. In the context of the retrospective estimation of reference limits, however, it is reasonable to assume that measurements taken from patients with specific diseases are differently distributed than those taken from patients considered healthy. If there is not enough information about the origin of the samples to either separate them before the analysis or adjust the model formulation accordingly, this latent heterogeneity means that the use of a single density function does not sufficiently describe the entire distribution of the analyte in the database. As a result, using the quantiles of the estimated distribution as reference limits would most likely overestimate the quantiles of the healthy population, as many pathologic measurements are either substantially higher or lower and thus tend to give more weight to one or both tails of the composite empirical distribution.

In these situations, given the number of components *M*, a weighted sum of $m=1,\ldots ,M$ probability density functions $f_{m}(x^{(i)},\boldsymbol{\theta }_m)$ with corresponding parameter vector $\boldsymbol{\theta }_m$ can be used to construct a finite mixture distribution

$$\begin{aligned} f(x^{(i)},\boldsymbol{\psi })=\sum _{{m=1}}^{M}\alpha _{m}f_{m} (x^{(i)}, \boldsymbol{\theta }_m), \end{aligned}$$

where $\boldsymbol{\psi }=(\alpha _{1},\ldots ,\alpha _{M}, \boldsymbol{\theta }_1,\ldots ,\boldsymbol{\theta }_{M})$ contains all unknown weights and parameters to be estimated. With $\alpha _{m}>0$ and $\sum _{{m=1}}^{M}\alpha _{m}=1$, $f(x^{(i)},\boldsymbol{\psi })$ is a convex combination of all $f_{m}(x^{(i)}, \boldsymbol{\theta }_m)$ and thus a probability density function itself.

Finite mixture distributions have quite a long history [19, 20], are comprehensively described in the statistical literature [21–23] and continue to provide useful approaches to a wide range of applications (e.g. [24, 25]). The most common strategy to estimate the unknown parameters is the expectation maximization algorithm [26]. A well known limitation of EM algorithms, however, is that they become easily trapped in local maxima [27]. Other limitations involve identifiability problems for certain mixtures of density functions or when the number of components is misspecified [28]. In order to address this issue, several approaches based on bootstrapping methods have been suggested [29, 30].

### Generalized additive models for location, scale and shape

While the assumed distribution of an outcome $\varvec{y}$ is mostly described by multiple parameters, the main focus of most regression problems lies on using a set of given input variables *X* to model only the mean. Generalized additive models for location, scale and shape (GAMLSS) [8] extend the original framework of generalized additive models (GAM) [31] by defining additional additive predictors to model the dependency on the covariates of up to four distribution parameters $\boldsymbol{\theta }_{k=1,\ldots ,4}$:

$$\begin{aligned} g_k\big(\theta _k^{(i)}\big) = \beta _{0k} + \sum _{j=1}^{p_k}h_{j\theta _k} (x_{kj}^{(i)}) = \eta _{\theta _k}^{(i)} \end{aligned}$$

Here, $g_k(\cdot )$ denotes a known monotonic link function and $\varvec{x}_{k1},\ldots ,\varvec{x}_{kp_k}$ the $p_k$ (possibly different) input variables for each predictor $\boldsymbol{\eta }_{\theta _k}$ with $h_{j\theta _k}(\cdot )$ describing the shape of the effect of $\varvec{x}_{kj}$ on $\boldsymbol{\theta }_k$. The four parameters are also referred to more specifically by $\boldsymbol{\theta }_k=(\boldsymbol{\mu }, \boldsymbol{\sigma },\boldsymbol{\nu },\boldsymbol{\tau })$. Set up properly, this model class therefore allows to model patterns in the data that are for instance responsible for differences in the residual variance of Gaussian regression or the dispersion parameters for negative binomial regression. The model parameters are usually estimated via (penalized) maximum likelihood by means of either a Newton–Raphson or Fisher scoring algorithm. Later, the use of gradient boosting algorithms was proposed to allow for inference in high-dimensional settings via implicit regularization [32].

### Latent class distributional regression

In order to be able to simultaneously address both highly dynamic dependence structures of the distribution of interest as well as the problem of unlabeled observations from possibly multiple sources, we propose to combine the GAMLSS framework with the concept of mixture models. With a pre-specified number of components *M*, this results in a latent class distributional regression model

$$\begin{aligned} f\left( y^{(i)}\big |\varvec{x}^{(i)},\boldsymbol{\alpha }, \boldsymbol{\xi }^{(i)}\right) =\sum _{{m=1}}^{M}\alpha _{m}f_{m} \left( y^{(i)}\big |\varvec{x}^{(i)},\boldsymbol{\theta }_{m}^{(i)}\right) , \end{aligned}$$

with mixture weights $\boldsymbol{\alpha }=(\alpha _{1},\ldots , \alpha _{M})$ and $\boldsymbol{\xi }^{(i)} =(\boldsymbol{\theta }_1^{(i)},\ldots ,\boldsymbol{\theta }_{M}^{(i)})$ containing all observation and component specific distribution parameters. A schematic overview of the steps performed for model estimation is shown in Algorithm 1. After providing initial values for all individual observation weights $w_{m}^{(i,0)}$, the algorithm starts by fitting suitable GAMLSS for all of the *M* components, with data each time re-weighted using the corresponding vector $\varvec{w}_{m}^{(0)}$. In the same step, the initial mixture weights $\alpha _{m}^{(0)}$ are computed. Subsequently, the parameters $\hat{\boldsymbol{\theta }}_{m}^{(i,0)}$ of the initial model fit are then used to derive updates for the observation weights by computing the likelihood for each observation to be part of the separate models. With new weights $w_{m}^{(i,1)}$ available, the data is obviously re-weighted differently and refitting the models for each component again leads to new sets of parameter estimates $\hat{\boldsymbol{\theta }}_{m}^{(i,1)}$. Cycling through these alternating updates steadily increases the log-likelihood of the full mixture model and eventually converges to a solution where additional iterations will not bring any substantial improvements.
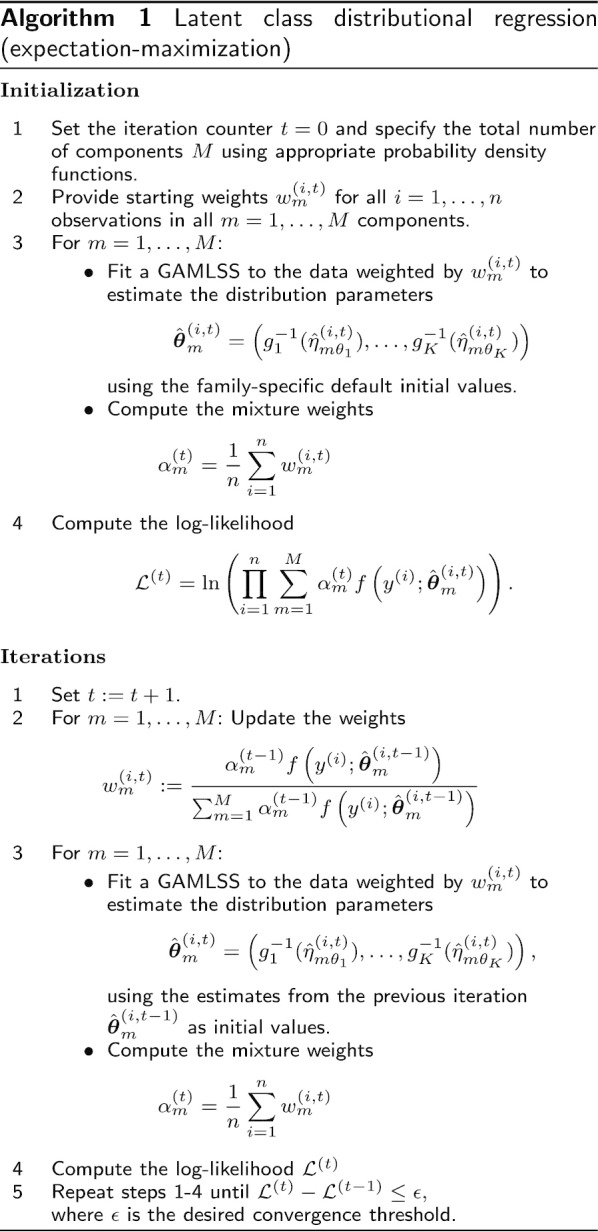


As mentioned in the introduction of mixture models, however, solutions found by the expectation-maximization approach may not always represent the global optimum. For example, unboundedness of the likelihood might occur at the edge of the parameter space, making the solution of the algorithm strongly depend on the provided initial observation weights [33]. Moreover, identifiability of standard linear regression models might not be guaranteed even if the regressor matrix has full rank [34].

## Data Availability

The R-code used to generate the datasets supporting the conclusions is available at https://doi.org/10.24433/CO.9246542.v3.

## Appendix

See Table 3.

**Table 3:** Number of non-converged runs (out of 1000) per setting for latent class distributional regression (LCDR)

*c*	*n* = 500			*n* = 1000			$\sum$
	$\alpha _1$			$\alpha _1$			
	0.6	0.7	0.8	0.6	0.7	0.8	
5	79	87	125	24	58	86	459
10	51	63	105	14	26	60	319
15	23	34	96	3	7	30	193
20	4	17	55	0	1	16	93
$\sum$	157	201	381	41	92	192	1064
